# Restoring Mandibular Anatomy After Complex Trauma: Clinical Applications of a Statistical Shape Model

**DOI:** 10.3390/jcm15031223

**Published:** 2026-02-04

**Authors:** Stephen A. L. Y. Youssef, Cornelis Klop, Juliana F. Sabelis, Ruud Schreurs, Jitske W. Nolte, Renee Helmers, Alfred G. Becking, Leander Dubois

**Affiliations:** 1 Department of Oral and Maxillofacial Surgery, Amsterdam UMC, University of Amsterdam, Meibergdreef 9, 1105 AZ Amsterdam, The Netherlands; c.klop@amsterdamumc.nl (C.K.); j.f.sabelis@amsterdamumc.nl (J.F.S.); r.schreurs@amsterdamumc.nl (R.S.); j.w.nolte@amsterdamumc.nl (J.W.N.); r.helmers@amsterdamumc.nl (R.H.); ag.becking@amsterdamumc.nl (A.G.B.); l.dubois@amsterdamumc.nl (L.D.); 2 Academic Centre for Dentistry Amsterdam (ACTA), University of Amsterdam and Vrije Universiteit Amsterdam, Gustav Mahlerlaan 3004, 1081 LA Amsterdam, The Netherlands

**Keywords:** mandibular reconstruction, maxillofacial injuries, computer-assisted surgery, three-dimensional imaging, statistical shape modeling, virtual surgical planning, patient-specific implants

## Abstract

**Background/Objectives:** Restoration of mandibular anatomy following complex trauma remains challenging when conventional anatomical and occlusal references, such as dental occlusion, contralateral morphology, condylar position, or mandibular continuity are lost. This technical note describes the clinical application of a mandibular statistical shape model as an alternative anatomical reference for diagnosis, virtual planning, and postoperative evaluation in patients with severe post-traumatic deformities. **Methods**: The MAGIC-SSM, an open-source, age-, and sex-specific three-dimensional model derived from a normative population dataset, enables reconstruction of plausible mandibular geometry in the absence of residual landmarks. Three clinical cases were analyzed using MAGIC-SSM-based reference alignment, with distance mapping applied when indicated. **Results**: The model provided an additional anatomical reference that supported decision-making in secondary correction, hybrid reconstruction with patient-specific implants, and quantitative evaluation of postoperative outcomes. **Conclusions**: By replacing lost spatial references with population-based geometry, the MAGIC-SSM offered support for restoring mandibular form and symmetry. These preliminary findings illustrate the feasibility of applying the MAGIC-SSM as an anatomical framework in complex trauma when conventional guides are absent. As its clinical application involved clinician-guided alignment and scaling, reproducibility and reliability remain to be established and require further validation.

## 1. Introduction

Surgical management of mandibular fractures is a foundational aspect of maxillofacial trauma care. For simple unilateral fractures, reduction and fixation are typically guided by stable anatomical references such as dental occlusion, contralateral bone morphology, and established mandibular biomechanics [1]. These reliable landmarks ensure predictable outcomes in cases where mandibular continuity is preserved [2].

In more complex injuries, such as comminuted, bilateral, or segmental fractures, these references are frequently disrupted or completely lost [3,4]. The absence of occlusal support, bilateral condylar involvement, or severe bone fragmentation deprives surgeons of critical spatial orientation. Both manual reduction and conventional digital planning become unreliable, increasing the risk of skeletal asymmetry, malocclusion, and condylar mispositioning [5,6,7].

Although virtual surgical planning (VSP) has significantly enhanced the precision of mandibular reconstructions in many scenarios, its effectiveness fundamentally relies on the presence of identifiable anatomical landmarks [8]. Conventional VSP strategies such as contralateral mirroring, occlusion-based alignment and pre-injury imaging are often inapplicable in high-complexity cases, even more in edentulous patients, bilateral comminution, or severe panfacial trauma [9,10,11]. When such patient-specific landmarks are absent, current VSP workflows do not provide an independent anatomical reference to support reconstructive decision-making. Pediatric and adolescent patients present additional challenges due to ongoing mandibular growth and development, further limiting the utility of adult-based templates or static anatomical references [12,13].

Klop et al. developed the MAGIC statistical shape model (MAGIC-SSM), an open-source, age-, and sex-specific three-dimensional model of mandibular growth to address these gaps [14]. Utilizing a large normative dataset, this model generates plausible mandibular geometries based on population-derived growth trajectories, independent of patient-specific anatomy or prior imaging. As a statistically derived anatomical reference, it extends the capabilities of existing planning methods in scenarios lacking conventional landmarks [15,16].

The original MAGIC-SSM publication focused on model development and validation within a normative context and did not evaluate its clinical application in mandibular trauma reconstruction. On this technical note, we present three clinical applications of the MAGIC-SSM in complex mandibular trauma, illustrating its role as an independent population-based anatomical reference across different stages of the reconstructive workflow, when conventional VSP strategies are not applicable. The aim of this technical note is to explore whether this population-based reference can meaningfully support reconstructive decision-making in severe traumatic deformities.

## 2. Materials and Methods

### 2.1. Construction of the MAGIC-SSM

The construction of the mandibular growth model has been described comprehensively in the original publication [14]. In summary, computed tomography (CT) scans of healthy children and young adults (0–22 years of age) were collected from various forensic and clinical databases. The mandible of each sample was segmented using a (semi)-automatic segmentation method. A template mandible was mapped onto each sample through rigid and non-rigid registration techniques to establish point correspondence across all cases. The resulting mappings were subsequently aligned using the Procrustes algorithm. The final growth model comprises 678 samples (66% male, 34% female). Associated metadata includes biological sex and chronological age. The growth model was validated for geometrical and anatomical correctness. All data is openly accessible on an online repository: DOI 10.5281/zenodo.8340160.

### 2.2. Application of the MAGIC-SSM

**Figure 1:**
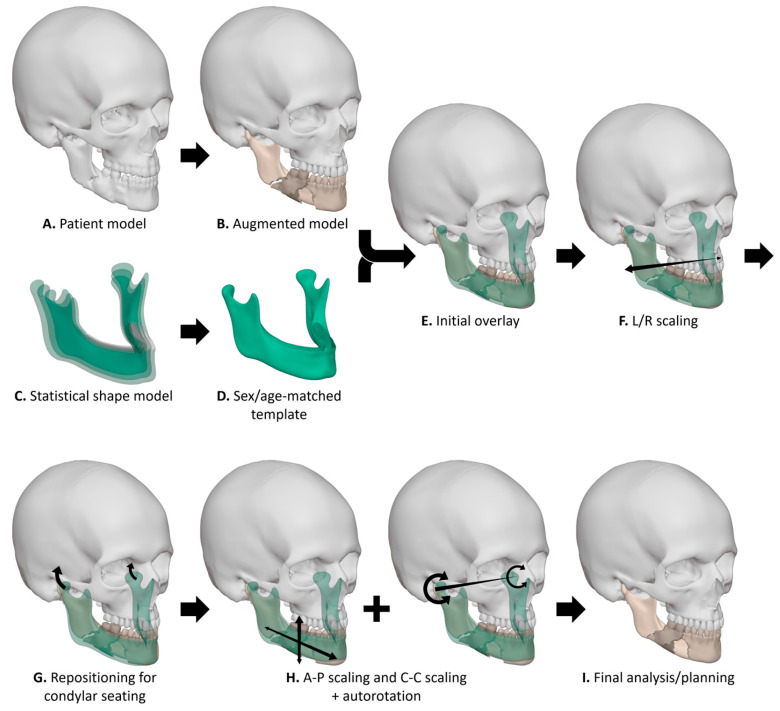
Workflow for application of the statistical shape model. The patient model (**A**) is used to construct an augmented model (**B**), which provides a clear overview of the patient’s anatomy and facial trauma. A sex- and age-matched template (**D**) is extracted from the statistical shape model (**C**) and superimposed onto the patient (**E**). The template is then scaled in lateromedial direction (**F**); repositioned to retruded contact position (**G**); and iteratively scaled in anteroposterior direction, scaled in craniocaudal direction, and autorotated along the condylar axis (**H**). These steps form the basis for the final analysis or treatment planning (**I**).

The workflow for application of the MAGIC-SSM is illustrated in Figure 1. For the case series presented in this study, segmentation was performed using IPS CaseDesigner (version 2.5, KLS Martin, Tuttlingen, Germany) or the Brainlab environment (Origin/iPlan versions 3.0–3.2, Brainlab AG, Munich, Germany). The virtual planning was performed by one of the clinical engineers in the department (C.K., J.F.S., or R.S.) and subsequently confirmed or adjusted by the surgeon (L.D.). The patient model (Figure 1A) was augmented to enable a clearer assessment of the mandibular bone and dental condition. The augmented patient model may include the virtual removal of osteosynthesis material, separation of bone segments, or fusion of an intra-oral scan (Figure 1B).

Ideally, a sex-matched and age-matched reference mandible would be generated for each patient using the aforementioned MAGIC-SSM. However, the patients included in this study exceeded the upper age limit of the samples used to construct the growth model. Based on longitudinal craniofacial data showing only small, gradual skeletal changes after late adolescence, we considered the first decades of adulthood morphometrically stable and used a 20–22-year reference for adult templating [17,18,19,20]. A reference mandible was constructed by averaging all male or female samples within the 20–22-year age bracket (Figure 1C,D). This yielded a reference mandible based on 75 samples for males and 25 samples for females.

Although the process of applying the MAGIC-SSM in a virtual planning is patient-specific and partially operator-dependent, several standardized steps were followed. Both the patient model and the reference model were imported into Blender (version 4.2, Blender Foundation, Amsterdam, The Netherlands). The reference model was initially aligned to the patient’s mandible using the iterative closest point (ICP) algorithm (Figure 1E). The dimensions of the reference mandible were adjusted to approximate the patient’s pretraumatic anatomy. The reference model was scaled along the left-right axis to match the mandibular fossae of the patient (Figure 1F). The mandible was repositioned until the condyles reached retruded contact position (RCP) (Figure 1G). The condylar seating of the reference mandible was verified by overlaying it onto the CT scan using IPS CaseDesigner or Brainlab Origin/iPlan. Additional scaling in anterio-posterior and cranio-caudal directions was performed to harmonize the bony profile and lower facial height, respectively (Figure 1H). This process was combined with autorotation of the mandible to adjust the intermaxillary distance (Figure 1H).

**Figure 2:**
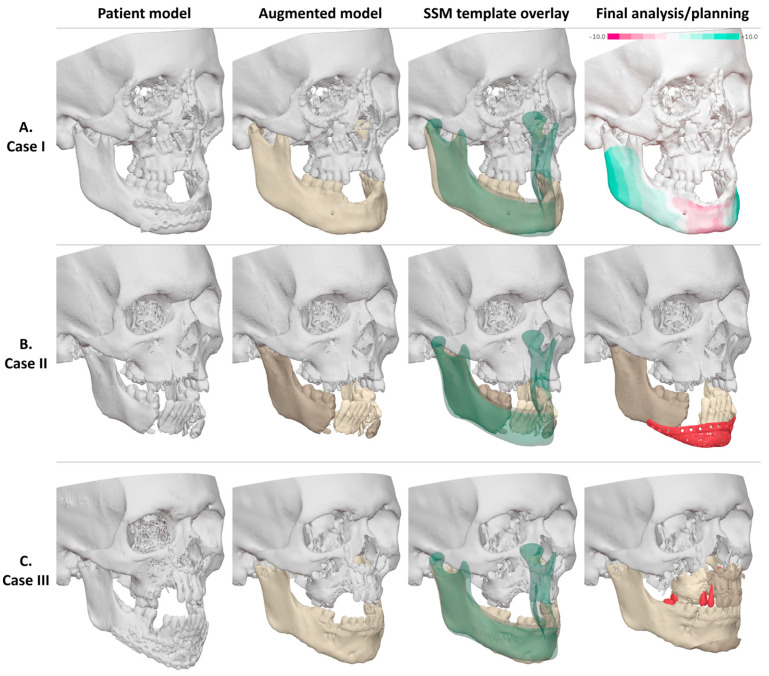
Virtual workup for the presented cases. The patient model, augmented model, template superimposition, and final analysis or treatment planning are shown for each case.

A distance map between the patient’s mandible and the reference mandible was generated in Blender to assess the extent and severity of anatomical deviations. The distance map was constructed by ray-casting a normal vector from the patient’s mandible to the reference model. An appropriate maximum distance was chosen (e.g., 10 mm), depending on the maximum anatomical deviation of the patient. A gradient colormap was used to visualize the severity of anatomical deviations on the patient’s mandible. Regions with a surplus compared to the reference model were visualized in green, and regions with insufficiency compared to the reference model were shown in red. Regions with appropriate projection were visualized in white. After discussing the extent and severity of the anatomical deviations with the surgeon, a patient-specific virtual treatment planning was created by the surgeon and clinical engineer. A treatment planning may include a mandibular osteotomy, repositioning of bone segments, or patient-specific implant (PSI) (Figure 1I and Figure 2).

Model performance was assessed by whether the MAGIC-SSM yielded anatomically coherent reference geometry that meaningfully supports diagnostic interpretation, virtual planning, or postoperative assessment in cases with compromised conventional landmarks.

## 3. Results

### 3.1. Case I: SSM-Guided Distance Mapping for Diagnosis and Staged Reconstruction

#### 3.1.1. Clinical Presentation

A 22-year-old male sustained severe craniofacial trauma in a snowmobile accident in Finland. The injury pattern consisted of a Le Fort III fracture on the right, a Le Fort II fracture on the left, a comminuted symphyseal mandibular fracture, a right naso-orbito-ethmoidal (NOE) fracture, and a right orbital floor fracture. Primary treatment abroad included open reduction and internal fixation of the comminuted symphyseal mandibular fracture, the right frontozygomatic buttress, the NOE fracture, the maxilla, and reconstruction of the right orbital floor. The patient was referred to the Department of Oral and Maxillofacial Surgery, Amsterdam UMC for tertiary reconstructive planning due to persistent orbital deformities (diplopia, enophthalmus, hypoglobus, and telecanthus) and midfacial asymmetry. The midfacial fractures altered maxillomandibular relations and thereby secondarily affected mandibular position. Additional findings during examination included a transverse maxillary deficiency, trismus, sensory disturbance, and facial nerve weakness.

#### 3.1.2. Morphological and SSM-Based Anatomical Assessment

CT imaging was analyzed using complementary virtual planning techniques. Distance mapping against the MAGIC-SSM-derived reference quantified regional mandibular deformations (Figure 2), providing an objective basis for identifying areas requiring surgical correction, including bilateral corpus–angle prominence of 9.3 mm on the left, 6.5 mm on the right, and a 6.9 mm symphyseal retrusion. The distance map further demonstrated that the anterior mandible was positioned below the normative vertical height, indicating the need for vertical augmentation to enable future implant-supported rehabilitation. Orthognathic analysis showed a skeletal class III relationship with maxillary retrusion, yaw, and roll malposition. For the midface and orbit, contralateral mirroring quantified right-sided deformity (infraorbital rim: +4 mm dorsal, +2 mm caudal; zygomatic arch: +3 mm widening) and an orbital volume increase of 6 cc, correlating with enophthalmos and hypoglobus due to the loss of infraorbital support.

#### 3.1.3. Impact on Virtual Surgical Planning

The distance mapping, orthognathic analysis, and contralateral mirroring together provided targets that governed the sequence, vectors, and magnitude of skeletal corrections, forming the basis of the virtual surgical plan. The MAGIC-SSM functioned as an independent mandibular reference in the absence of reliable occlusal or contralateral landmarks, allowing for the objective assessment of mandibular projection, vertical height, and symmetry.

#### 3.1.4. Planned Surgical Reconstruction

A staged approach was adopted. The first stage consisted of correction of midfacial asymmetry and orbital deformity through a right zygomatic osteotomy, NOE osteotomy, and three-wall orbital reconstruction. The second stage comprised bimaxillary osteotomies with vertical augmentation of the anterior mandible, guided by the quantitative mandibular discrepancies indicated by the distance map.

#### 3.1.5. Surgery and Postoperative Evaluation

At the time of reporting, staged reconstruction had been completed according to the virtual plan, with postoperative evaluation focusing on correction of mandibular symmetry, midfacial alignment, and restoration of orbital volume.

### 3.2. Case II: SSM-Guided Segment Preservation, PSI Design, and Reconstruction

#### 3.2.1. Clinical Presentation

A 27-year-old male was referred to the Department of Oral and Maxillofacial Surgery, Amsterdam UMC for secondary reconstruction following severe craniofacial trauma sustained in a fall from height two months earlier. CT imaging demonstrated bilateral condylar head fractures with medial displacement and a severely comminuted symphyseal and paramedian mandibular fracture with partial avulsion and loss of the inferior border. No acute maxillofacial intervention was performed at the referring hospital, as other injuries required prioritization.

#### 3.2.2. Morphological and SSM-Based Anatomical Assessment

Radiographic assessment included panoramic radiography and CT imaging, complemented by intraoral scanning, standardized photography, and three-dimensional facial imaging. Imaging confirmed bilateral condylar head fractures with medial displacement and a severely comminuted symphyseal and paramedian mandibular fracture with partial avulsion and loss of the inferior border. Mandibular morphology was analyzed using the MAGIC-SSM (Figure 2), demonstrating that despite severe comminution and inferior border loss, the remaining anterior mandibular segment was within the lower normative range for symphyseal height and projection. Preservation of this segment was therefore considered feasible, while the discontinuity defect measuring approximately 27 mm in length, 20 mm in vertical height, and 12 mm in anteroposterior dimension required prosthetic reconstruction.

#### 3.2.3. Impact on Virtual Surgical Planning

Reconstructive options were evaluated. A free fibula flap was considered to restore continuity but deferred due to the overall treatment burden in the context of psychiatric comorbidities. The MAGIC-SSM-supported assessment justified a hybrid strategy consisting of preservation of the viable anterior segment combined with PSI reconstruction to restore the mandibular contour and continuity. The PSI design was guided by the normative mandibular morphology derived from the MAGIC-SSM. Screw positioning was optimized during planning for bicortical locking fixation with 11 mm screws in the anterior segment and 12 mm screws in the left ramus. The implant consisted of a 2 mm titanium baseplate integrated with a 1 mm lattice framework to serve as a scaffold for particulate bone graft, allowing combined rigid fixation and biological augmentation. Prosthetic-driven planning ensured adequate alveolar width and intermaxillary relationships for future implant rehabilitation.

#### 3.2.4. Planned Surgical Reconstruction

Mandibular reconstruction via a submandibular approach was planned, with exposure of the mandibular defect; adaptation of the PSI to the residual anterior segment and left ramus; and filling of the lattice structure with particulate bone graft to restore vertical height, mandibular projection, and alveolar volume.

#### 3.2.5. Surgery and Postoperative Evaluation

The patient underwent mandibular reconstruction as planned. Minimal contouring of the left ramus (<1 mm) was required to allow intimate adaptation of the PSI. Bicortical locking fixation was achieved, and particulate bone grafting was performed to restore mandibular height, contour, and chin projection. These steps followed established principles for patient-specific alloplastic mandibular reconstruction [21]. Primary closure was tension-free, and no intraoperative complications occurred.

### 3.3. Case III: Diagnostic Application for Secondary Reconstruction

#### 3.3.1. Clinical Presentation

A 23-year-old male sustained severe craniofacial trauma in September 2024 following a fall from height. Initial treatment performed abroad consisted of open reduction and internal fixation of a left mandibular condylar neck fracture, bilateral mandibular body fractures, and a premaxillary fracture. In January 2025, he was referred to the Department of Oral and Maxillofacial Surgery, Amsterdam UMC for secondary rehabilitation. Clinical examination revealed a maxillomandibular midline discrepancy with an associated chin deviation. An anterior open bite was accompanied by reduced right laterotrusion, occlusal disturbances, and mutilated dentition.

#### 3.3.2. Morphological and SSM-Based Anatomical Assessment

Radiographic evaluation including panoramic radiography and CT imaging confirmed the postoperative situation following fixation of complex mandibular and maxillary fractures. Intraoral scanning, standardized photography, and three-dimensional facial imaging were performed to support virtual analysis. Mandibular anatomy was evaluated using the MAGIC-SSM as an independent anatomical reference (Figure 2). The reconstruction was considered largely acceptable relative to normative mandibular morphology, with mild underprojection of the chin region contributing to facial asymmetry and midline deviation. No major transverse or vertical discrepancies of the mandibular body were identified.

#### 3.3.3. Impact on Virtual Surgical Planning

The MAGIC-SSM-based assessment supported a conservative mandibular strategy, indicating that an extensive mandibular re-osteotomy was not required. Removal of existing mandibular osteosynthesis material was anticipated as a preparatory step. MAGIC-SSM analysis informed the decision to focus correction on maxillary repositioning combined with targeted genioplasty rather than comprehensive mandibular reconstruction.

#### 3.3.4. Planned Surgical Reconstruction

The virtual surgical plan comprised a three-piece Le Fort I osteotomy with 6.5 mm maxillary advancement, 6.0 mm anterior impaction, and a 3.0 mm midline shift to the left to improve dental show and reduce anterior diastemas. This was combined with a sliding genioplasty with 6.0 mm advancement and a 1.5 mm midline correction to the left. Orthodontic planning included extrusion of third molars, mesialization of posterior mandibular teeth, and lateralization of the mandibular anterior segment, followed by implant-based prosthetic rehabilitation.

#### 3.3.5. Surgery and Postoperative Evaluation

At the time of reporting, the patient was in the orthodontic preparation phase following removal of mandibular osteosynthesis material and placement of orthodontic anchorage devices. Definitive surgical correction had not yet been performed.

## 4. Discussion

The presented cases illustrate that the MAGIC-SSM provides an alternative anatomical framework in complex trauma. In all three cases, the MAGIC-SSM provided population-based normative guidance, enabling objective assessment of mandibular discrepancies, alignment, and projection. In the first case, it quantified deformities and informed osteotomy trajectories despite loss of dentition and contralateral landmarks. In the second, it supported preservation of mandibular segments and guided patient-specific implant design, avoiding the need for a free fibula flap. In the third, it enabled evaluation of a post-traumatic malunion, confirming existing reconstruction accuracy and directing surgical planning toward targeted maxillary and genioplasty corrections. Across all scenarios, the MAGIC-SSM added critical value by supplying reliable anatomical reference where conventional landmarks were unavailable or unreliable, transforming complex cases into precise, population-informed surgical plans.

The presented cases demonstrate that the MAGIC-SSM can be applied for different purposes across the treatment spectrum: as a diagnostic tool to evaluate existing reconstructions and identify corrective vectors, as a preoperative planning instrument guiding complex reconstructive strategies including PSI design, and as a postoperative reference to quantify outcomes against normative references. This breadth of application underscores the versatility of the SMM and its potential to integrate consistently into multiple stages of maxillofacial trauma care.

Compared to traditional planning strategies, the potential role of an SSM becomes apparent. Contralateral mirroring is well established for unilateral injuries but cannot be applied when both sides are involved or asymmetrically healed [10,11,12]. Occlusion-driven planning remains a cornerstone of mandibular reconstruction but is not feasible in edentulous patients and unreliable in cases of unstable occlusion [1,6,7]. Free fibula flap reconstruction remains the standard approach for continuity defects [22]; however, the use of an SSM may support consideration of alternative strategies in selected situations [23].

While our series illustrates these trauma-specific applications, previous studies on SSMs have primarily focused on reconstructing missing bony geometry or guiding orthognathic planning. Several groups have demonstrated that SSMs can reliably predict mandibular morphology from partial segments with validated geometric accuracy, allowing for virtual reconstruction of continuity defects or asymmetric growth patterns [23]. These approaches were largely validated in controlled experimental or orthognathic cohorts, showing that population-derived templates can approximate missing anatomy with high accuracy. SSM-tools have been incorporated into craniofacial planning workflows as open-source applications, supporting their potential to serve as anatomical references independent of contralateral mirroring or occlusion-based strategies [14,16,23,24].

In contrast to these earlier studies, which focused on elective orthognathic surgery or developmental anomalies, our work demonstrates the applicability of an SSM in high-complexity trauma. Previous reports on PSI-based reconstructions with or without bone grafts highlight potential alternatives to vascularized free flap transfer in selected patients [9,10,11], but they lack an independent anatomical reference when local landmarks are destroyed. By integrating the MAGIC-SSM into trauma workflows, we explored the application of the SSM beyond elective deformity correction, including acute and secondary fracture management. This comparison highlights that while earlier SSM literature validated the concept in relatively controlled conditions, our cases illustrate its value in the most challenging reconstructive scenarios, where it complements conventional planning methods and expands the armamentarium of mandibular trauma surgery.

Several limitations deserve emphasis. The current MAGIC-SSM is confined to the mandibular skeleton and does not incorporate dentition, occlusal function, or soft-tissue relationships. Its normative dataset, though extensive, may not fully capture ethnic diversity or extremes of mandibular morphology, and validation in older or edentulous patients is limited [14]. Furthermore, the reference models derived from the MAGIC-SSM did not strictly correspond to the age of two patients in this study, since reference cases within the 20–22-year age bracket were used for patients in their late twenties. This approach was considered acceptable, as the mandible is found to be morphometrically stable during the first decades of adulthood [17,18,19,20]. Additionally, the application of the MAGIC-SSM in virtual trauma planning is presumably operator-dependent, although several standardized steps were followed. This technical note describes a small series of selected cases and lacks a control group, precluding definitive conclusions about comparative effectiveness or long-term outcomes.

Several practical pitfalls related to its clinical use should be recognized. The MAGIC-SSM should not be interpreted as a stand-alone decision-making tool or as a replacement for established reconstructive strategies but rather as an adjunctive anatomical reference to support surgical planning when conventional landmarks are absent or unreliable. Misapplication may occur if population-based reference geometry is interpreted as patient-specific anatomy without appropriate clinical judgment, particularly in complex trauma cases requiring individualized biomechanical or functional considerations [25]. In addition, reliance on the model without critical assessment of residual segment positioning and fracture biology may lead to inappropriate planning assumptions, which may contribute to inadequate stabilization and impaired fracture healing [4]. To avoid these pitfalls, use of the MAGIC-SSM should remain embedded within a multidisciplinary planning process and combined with established reconstructive principles, surgeon expertise, and case-specific clinical priorities.

## 5. Conclusions

For the MAGIC-SSM to be meaningfully integrated into trauma care, prospective validation studies comparing SSM–guided reconstructions with conventional approaches are essential. Such studies should evaluate not only anatomical accuracy but also functional outcomes and patient-reported results, to establish its clinical utility. Its value would be further enhanced if the cranium and dentition could also be incorporated into the model—an extension of the MAGIC-SSM that is currently under development. Within the context of this technical note comprising three cases, these experiences indicate that the MAGIC-SSM, although conceived as a growth reference, can also be repurposed as a normative framework in adult trauma. By providing a reproducible, population-based anatomical template in situations where conventional references are lost, the MAGIC-SSM has the potential to complement existing reconstructive strategies and facilitate more consistent, data-driven decision-making in complex mandibular trauma.

## Data Availability

The MAGIC-SSM model is openly accessible on an online repository: DOI 10.5281/zenodo.8340160.

## Abbreviations

CT	Computed tomography
ICP	Iterative closest point
MAGIC-SSM	Mandibular Statistical Shape Growth Model
NOE	Naso-orbito-ethmoidal
ORIF	Open reduction and internal fixation
PSI	Patient-specific implant
RCP	Retruded contact position
SSM	Statistical shape model
VSP	Virtual surgical planning
